# Supplementary material for: The Evaluation of Radiolabeled Prostate-Specific Membrane Antigen Positron Emission Tomography/Computed Tomography for Initial Staging in Intermediate-Risk Prostate Cancer Patients: A Retrospective Multicenter Analysis
Source: Diagnostics (Basel). 2024 Dec 6;14(23):2751. doi: 10.3390/diagnostics14232751 (PMC11640276; doi:10.3390/diagnostics14232751)
Supplement: Supplementary file 1 [file diagnostics-14-02751-s001.zip › diagnostics-3328789-supplementary.pdf]

Supplemental Table S1. Synthesis and quality control for the PSMA tracers' production in each center.

Center	[18F]F-PSMA-1007	[68Ga]Ga-PSMA-11
Humanitas Research Hospital, Rozzano, Italy	• Trasis ALLINONE-18 Synthesizer<br>• Trasis ALLINONE-36 Synthesizer	• Trasis MiniAllInOne Synthesizer
Humanitas Gavazzeni, Bergamo, Italy	-	• Trasis EASY ONE Synthesizer
Humanitas Istituto Clinico Catanese, Catania, Italy	• Trasis ALLINONE-36 Synthesizer	• Trasis MiniAllInOne Synthesizer

Supplemental Table S2. PET scanners in each center

Center	PET scanner	Accreditation system (year)
Humanitas Research Hospital, Rozzano, Italy	• PET/CT System 1: GE Discovery D690 ToF (CT slice: 8; method of reconstruction: TOF, VPHD, 256x256)<br>• PET/CT System 2: Siemens Biograph Vision 600 (methods of reconstruction: TOF, 256x256)	• EARL accreditation standard 1: 18F (2024)<br>• EARL accreditation standard 2: 18F (2024)
Humanitas Gavazzeni, Bergamo, Italy	• PET/CT System: GE Discovery IQ (CT slice: 16; method of reconstruction: PSF, QCHD, VUE Point, 256x256)	• EARL accreditation standard 2: 18F (2024)
Humanitas Istituto Clinico Catanese, Catania, Italy	• PET/CT System 1: GE Discovery D690 ToF (CT slice: 8; method of reconstruction: TOF, VPHD, 256x256)<br>• PET/CT System 2: GE Discovery IQ (CT slice: 16; method of reconstruction: PSF, QCHD, 256x256)	• EARL accreditation standard 2: 18F e 68Ga (2024)<br>• EARL accreditation standard 2: 18F e 68Ga (2024)
